# Testis-sparing surgery for benign and malignant tumors: A critical analysis of the literature

**DOI:** 10.4103/0970-1591.44249

**Published:** 2008

**Authors:** Gianluca Giannarini, Andrea Mogorovich, Irene Bardelli, Francesca Manassero, Cesare Selli

**Affiliations:** Department of Urology, University of Pisa, Pisa, Italy

**Keywords:** Conservative surgery, frozen sections, germ cell and embryonal, Leydig cell tumour, neoplasms, testis, testicular neoplasms, ultrasonography

## Abstract

In order to explore the latest advances in organ-sparing treatment of testicular tumors, a literature search of the Medline/PubMed database was carried out for published data in the English language up to 2007.

In the recent past the management of testicular tumors has evolved in favor of a testis-sparing approach in selected cases, both in the adult and pediatric population. The widespread use of high-frequency testicular ultrasound has led to detecting an increasing number of asymptomatic, non-palpable, small-volume masses. A higher proportion of testicular lesions of benign nature than previously reported has now been documented. The high accuracy of frozen section examination and the increasing interest in the potential functional, psychological and cosmetic advantages related to preserved testicular parenchyma are other arguments currently favoring the adoption of an organ-sparing policy for most testicular masses. Greater experience has been gained in also managing conservatively malignant tumors. Patients with germ-cell cancer in solitary testis or bilateral tumors can be submitted to testis-sparing surgery, provided that the maximum size of the lesion is <2 cm, preoperative testosterone is normal and adjuvant radiotherapy of the residual parenchyma is delivered. Cancer-specific survival is excellent, local recurrence rate very low and androgen supplementation unlikely.

## INTRODUCTION

Modern oncology has incorporated the principle of organ preservation into the therapeutical armamentarium of various specialties, breast cancer being the paradigm for this strategy. The greatest advantage is a significant reduction in physical, functional and psychological short- and long-term morbidity related to conventional extirpative surgery, provided that the main intent remains not to jeopardize cancer control.

Increasing awareness of the advantages related to organ preservation has also been achieved in the recent past for non-malignant conditions affecting organs deputed to functions which deserve to be maintained, such as the kidney and liver.

Similar to what has happened for other cancers and conditions in urology, the management of tumors of the testis has evolved in the last decade in favor of an organ-sparing approach in selected cases. Until the late ’80s the axiom existed according to which testes bearing any suspicious mass had to be removed, based on the historically reported very low prevalence of lesions of benign nature (approximately 1%) and the belief that intraoperative biopsies in presence of malignancy would have invariably led to tumor seeding and disease progression.[1]

Conversely, a higher proportion than previously described of histologically proven benign testicular lesions has been recognized in recent years, and the widespread use of testicular ultrasound for various indications has led to detecting an increasing number of asymptomatic, non-palpable, small-volume masses.[2–4] The question has therefore emerged, whether the entire testis needs to be always sacrificed when a mass of unknown origin is diagnosed, even in the presence of a normal contralateral gonad. The high accuracy achieved by frozen section examination (FSE) in identifying both benign and malignant lesions, and increasing attention to the cosmetic, functional and psychological outcome of patients with testicular tumors are additional strong arguments currently favoring an organ-sparing approach.

In the present review we discuss the latest advances in the organ-sparing management of testicular tumors of different histology.

A comprehensive literature review using the Medline/PubMed database for full-length papers was performed up to December 2007 through a free text search strategy that included the following entry terms: “testi* tumour OR neoplasm”, “testi* sparing OR organ sparing surgery” and “conservative surgery”. The following limitations were applied to restrict the search: male, humans, English language, title/abstract. Two authors (GG and AM) reviewed the abstracts of the retrieved records and selected only those pertinent to the objectives of the present review. Finally, the corresponding articles were carefully examined and referenced papers of interest eventually retrieved. Only those articles reporting complete data with clinical relevance for the present review were considered.

In addition, published abstracts at international urological meetings (American Urological Association, European Association of Urology, European Society for Sexual Medicine, International Society for Sexual Medicine, Société Internationale d’Urologie) in the last decade were also critically examined, and considered for the present review only if of outstanding clinical significance.

All the papers were ranked according to the level of evidence of the Oxford Centre for Evidence-Based Medicine.

From all the material retrieved, 49 relevant full-length papers and one congress abstract were selected. Due to the very low incidence of testicular tumors, no randomized controlled trials comparing testis-sparing surgery (TSS) with radical orchidectomy (RO) exist, only case reports and retrospective outcome studies are available (maximum level of evidence 2c). For the purpose of the present review, we grouped the results of our search according to the issues that follow.

## TESTIS-SPARING SURGERY

### A) Operative technique

The first description of the operative technique was made in 1986 by Stoll *et al*.,[5] who described the use of high-frequency ultrasound as a guide to enucleate a non-palpable Leydig cell tumor, and progressively developed until 2002, when Hopps and Goldstein[6] codified the procedure introducing the use of a magnification system, with the aim of improving the identification and complete excision of small non-palpable lesions.

**Figure 1:**
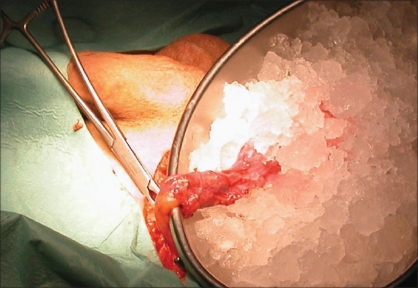
After the spermatic cord has been clamped, the testis is immerged in ice slush solution, the mass is removed and cooling is protracted until the report of frozen section examination is available

**Figure 2:**
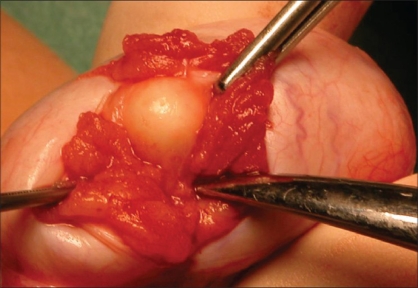
The mass is easily visualized by gently displacing the normal-looking surrounding testicular parenchyma with a small forceps. In the present case, a diagnosis of epidermoid cyst was made on frozen section examination and subsequently confirmed on permanent histology

**Figure 3:**
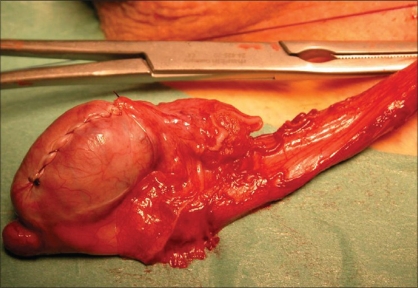
The integrity of the tunica albuginea is restored with a running 4-0 absorbable suture, after frozen section examination has diagnosed a Leydig cell tumor, the vascular clamp has been removed and complete hemostasis has been achieved

As a rule, the testis is delivered through a standard inguinal incision in preparation for RO. The spermatic cord is isolated, suspended and preventively clamped with a soft vascular clamp or occluded with a tourniquet. The gonad is then exteriorized from the same access and placed in a separate operative field, consisting of a folded towel resting on the ipsilateral upper thigh, to avoid potential spillage and wound contamination in case a malignant tumor is encountered. The gubernaculums testis is either clamped or sectioned. When cooling of the testis is performed, the testis is immerged in ice slush solution for 10 min after cord clamping and then kept in the same environment throughout the procedure so that a temperature of 15-19 °C is obtained [Figure 1]. The tunica vaginalis is opened and the testis inspected. An operating microscope, providing 6x to 25x magnification, can be used to aid identifying, and subsequently avoiding, the blood vessels subjacent to the tunica albuginea. The mass is localized by intraoperative ultrasound and a small-caliber needle may be placed adjacent to the lesion. The tunica albuginea overlying the tumor is then transversally or longitudinally incised and the mass visualized by gently displacing the parenchyma [Figure 2]. The lesion is enucleated leaving a 2-5 mm rim of normal-appearing testicular parenchyma around it and sent for FSE. Post-excision ultrasound can be used to show the complete removal of the mass. If pathological findings are benign, the testis and wound are irrigated with sterile water, the vascular clamp on the spermatic cord is removed, and after achieving complete hemostasis, the tunica albuginea is closed with running 4-0 or 5-0 absorbable suture [Figure 3]. If pathological findings are malignant, but RO is not performed (bilateral tumors or tumor in solitary testis), care must be taken to obtain multiple biopsies of the remaining parenchyma to rule out concomitant foci of malignancy or testicular intratubular germ cell neoplasia (TIN) on permanent histology.

### B) Role of frozen section examination

Frozen section examination is largely recognized as a key point in TSS. From a theoretical standpoint, TSS would be the ideal treatment for testicular masses, if intraoperative FSE could provide a diagnosis of their nature with absolute certainty.

Despite initial concern due to potential sampling error and insufficient quality of frozen section preparation,[7] FSE has recently demonstrated to be a highly reliable method to characterize testicular masses. Tokuc *et al*.[7] and Elert *et al*.,[8] reported that FSE was able to identify all malignant and benign testicular masses among 26 and 354 cases, respectively. Likewise, Leroy *et al*. reported a sensitivity of 81% for benign and of 100% for malignant lesions in 15 patients[9] and Connolly *et al*., a 94.2% positive predictive value and a 92.6% negative predictive value for malignancy in 80 patients.[10] Among malignant tumors, an approximately 10% failure to differentiate seminomatous from non-seminomatous forms has been documented,[8] but this has no consequences on the type of surgical procedure to be performed.

Furthermore, non-conclusive diagnosis on FSE has been only rarely observed.[6,11]

The main current criticism relates to the fact that these high figures in FSE accuracy may be related to the expertise of the attending dedicated uropathologist, which cannot be translated to every community hospital. This potential pitfall needs to be addressed in further investigations.

### C) Functional outcome after testis-sparing surgery

Given that a non-negligible proportion of patients with testicular tumors of all dignities are infertile, preservation of testicular parenchyma may be of paramount importance from a functional standpoint and may sensibly reduce the psychosocial consequences related to RO.[12] Additionally, it must be considered that young men undergoing unilateral RO for malignant tumors are likely to experience late onset hypogonadism,[13] albeit further research is needed to ascertain whether this applies also to patients whose testes are removed for a benign lesion.

However, major concerns on the potential advantages of TSS have been raised as far as patients with malignant germ cell tumors are regarded, since these are prone to functional testicular impairment (i.e. endocrine function and fertility) due to a number of reasons:

i) Testicular ischemia during spermatic cord clamping has the potential to impair endocrine and exocrine function. While this is incontrovertibly true for long-lasting impaired blood supply (i.e. prolonged acute testicular torsion),[14] sufficient experimental and clinical evidence suggests that no irreversible damage occurs, even under warm ischemia, provided that the spermatic cord clamping time does not exceed 30 min.[15,16] This time span has been shown to be reasonable to perform TSS with FSE.[10,17]

ii) Radiotherapy to eradicate concomitant TIN in the ipsilateral testis inevitably leads to the arrest of spermatogenesis. This, however, can be safely postponed, if fathering a child is planned.[16] In any case, patients should be counseled as to sperm donation or testicular sperm extraction and preservation.

iii) Radiation therapy was also shown to deteriorate Leydig cell function, which may be impaired as a preexisting abnormality in these patients. Accordingly, patients with baseline androgen deficiency are usually excluded from this approach.

iv) Recent findings have shown that TSS does not seem to negatively affect the possibility to father children, although violation of the tunica albuginea could theoretically induce the production of antisperm antibodies causing autoimmune infertility by altering the blood-testis barrier. Leonhartsberger *et al*.,[18] found comparable postoperative levels of antiserum sperm antibodies in two groups of patients submitted either to RO or TSS for malignant testicular tumors. Consistently, data extracted from the study of Giannarini *et al*.,[17] on TSS in patients with Leydig cell tumors show a preservation of fertility in terms of paternity after organ-sparing surgery.

Admittedly, these data are clearly not conclusive, and their applicability to all patients with benign testicular lesions is not established so far. From a functional standpoint, both, the proportion of patients who would truly benefit from a curative organ-sparing approach and the length of time this advantage would last remain to be determined.

### D) Indications for testis-sparing surgery

#### 1. Benign testicular tumors

The undisputed indication for TSS is a histologically confirmed benign lesion. All the above enumerated advantages related to the preservation of testicular parenchyma are, in fact, maximally exploited in this context.

In a comprehensive review including their own experience, Heidenreich *et al*.,[19] described the use of organ-sparing treatment in more than 120 patients with epidermoid cyst of the testis. After a follow-up of up to 37 years, no single patient suffered from local recurrence or distant metastases.

Several studies are available, which report on the medium- and long-term outcome of patients submitted to TSS for Leydig cell tumor, which exhibits a benign behavior with only a minority of metastasizing cases mostly confined to older reports.[17] The high number of articles reporting on TSS for this disease entity probably reflects the fact that, compared to most testicular lesions, Leydig cell tumor may be suspected from typical symptoms/signs (gynecomastia, infertility, endocrine abnormalities) or ultrasound characteristics.[17,20] A total of over 100 patients with Leydig cell tumor treated with TSS have been reported in the literature. The results of the two largest contemporary series of men electively operated on with testis preservation have been recently presented by Giannarini *et al*.[17] and Carmignani *et al*.[21] After a mean follow-up of approximately eight and four years, respectively, no patients experienced local or distant recurrence. The single case of local recurrence of Leydig cell tumor has been reported by Wegner *et al*.[22] and occurred six months after TSS despite the small size of the lesion and negative surgical margins.

Other lesions with incontrovertibly or prevalently benign nature for which TSS was carried out are cyst of the tunica albuginea or intraparenchymal simple cyst, dermoid cyst, adenomatoid tumor, inflammatory pseudotumor, post-inflammatory fibrosis, granulomatous flogosis, hemangioma, and Sertoli cell tumor.[2,3,8,23] All these lesions were reported to pose no interpretative doubts on FSE.

#### 2. Malignant testicular tumors

The first description of TSS for a malignancy was reported by Richie,[24] who performed a hemi-orchidectomy in a patient with bilateral seminoma. Although infertile, the patient remained free of disease at the 2.5-year-follow-up with no need of androgen supplementation. This approach was labeled as “unorthodox” by the author himself, but stimulated the research of other investigators. Since then, in fact, several series of TSS for imperative indications (synchronous or metachronous bilateral tumors or tumor in solitary testis) have appeared in the literature.[25]

Based on the pioneering work of the German researchers Heidenreich *et al.*[1] and Weissbach,[26] the German Testicular Cancer Study Group has gained leading international experience in this field and have recently presented the updated results.[27] Indications and recommendations for TSS in malignant lesions are the following: 1) tumor in solitary testis or bilateral tumors 2) diameter of the lesion less than 2 cm 3) no invasion of the rete testis 4) multiple biopsies of the surgical bed 5) adjuvant radiotherapy to the remaining testicular parenchyma to eradicate concomitant TIN 6) normal preoperative serum LH and testosterone levels 7) patient’s compliance to meticulous follow-up. Adhering to these principles has resulted in excellent long-term cancer-specific survival (nearly 100%), low local recurrence rate (4%) and normally preserved testosterone levels in the vast majority of patients (>83%).

No description of elective (i.e. with a normal contralateral testis) enucleation of a malignant tumor has appeared in the literature reported so far, possibly due to the reported almost universal presence of TIN or distant foci of malignancy in the surrounding testicular parenchyma, which would lead to local recurrence if not otherwise treated.[28]

#### 3. Non-palpable testicular tumors

Several series are now available, reporting on the management of patients diagnosed with asymptomatic, non-palpable and small-volume masses incidentally detected at scrotal ultrasound performed for various reasons and in whom either TSS or RO were adopted[4,6,9,12,29–34] [Table 1].

**Table 1:** Published series reporting the adoption of organ-sparing surgery and/or radical orchidectomy for the management of adult non-palpable testicular tumors

Author	Year	n° patients	size range(mm)	n° imperative TSS	n° elective TSS	n° RO	n° other therapy	n° benign FSE	n° malignant FSE	n° non conclusive FSE	n° benign FINAL	n° malignant FINAL	n° non available FINAL
Buckspan	1989	4	3-6	0	4	0	0	4	0	0	4	0	0
Hopps	2002	4	2-16	1	3	0	0	3	0	1	2	2	0
Carmignani	2003	10	4-16	0	7	3	0	10	0	0	10	0	0
Leroy	2003	15	4-16	0	9	6	0	9	4	2	11	4	0
Sheynkin	2004	9	NR	0	1	7	1*	1	0	0	6	2	1
Carmignani	2004	3	NR	0	3	0	0	3	0	0	3	0	0
Colpi	2005	5	3-5	0	4	1	0	4	1	0	4	1	0
Rolle	2006	7	2-16	0	6	1	0	6	1‡	0	6	1	0
Assaf	2006	6	4-20	1	1	2	2†	1	1	0	2	2	2
Müller	2006	20	1-5	0	16	4	0	17	3	0	16	4	0
Powell	2006	4	5-6	0	4	0	0	4	0	0	2	2	0

TSS - testis-sparing surgery; RO - radical orchidectomy; FSE - frozen section examination; FINAL - permanent histology; NR - not reported

* refused surgery, lost to follow-up

† active surveillance

‡ testicular intraepithelial neoplasia

The majority of these studies showed that, compared to palpable testicular lesions which are malignant in over 90% of cases,[9,11] in these patients a prevalence of benign tumors has to be expected. In a series of 27 patients with ultrasound-detected testicular lesions, Carmignani *et al*.,[4] reported an overall 51.8% prevalence of benign disease at definitive histology, with 80% of non-palpable lesions being benign. Similarly, Sheynkin *et al*.,[30] reported a 75% prevalence of benign lesions among eight non-palpable testicular masses. It has also been shown that smaller lesions (<2 cm) are more likely to be benign.[10,35] It is important to note that up to 100% of non-palpable testicular lesions are Leydig cell tumors,[11] which are known to have a benign behavior, especially if small in size and occurring in young individuals.[36]

It is of note that, as shown in Table 1, nearly all patients included in this category of testicular lesions in which TSS was performed, had an elective indication for surgery, that is a mass in presence of a healthy contralateral testis. Thus, albeit not formally established as standard treatment yet, TSS should be considered as a viable option for all cases of non-palpable tumors, particularly of small size, with the result of sparing an unnecessary RO in over half of patients whose testes do not bear cancer.

#### 4. Testicular tumors in the pediatric age

Considerable experience has been achieved also in the pediatric population, which would theoretically profit the most from an organ-sparing approach. In fact, compared to the adult age scenario, there is a higher incidence of benign testicular lesions, and malignant cases are not frequently associated with concomitant TIN or distant metastases.[37,38] Furthermore, fertility and semen quality have been found to correlate well with testis volume, which remains stable within the range of normality in the long term following TSS.[39] Finally, potential psychological and cosmetic advantages for the developing child are evident when testicular parenchyma is retained.

The first report dates back to 1983, when Marshall *et al*.,[40] reported on one out of eight benign lesions (namely a cystic teratoma) managed with TSS without recurrence at 1.5-year follow-up. In Table 2 the contemporary series reporting the use of TSS in children with testicular masses are presented.[41–47]

**Table 2:** Contemporary published series reporting the adoption of organ-sparing surgery and/or radical orchidectomy for the management of testicular tumors in the pediatric age

Author	Year	n° patients	n° imperative TSS	n° elective TSS	n° RO	n° other therapy	diagnosis FSE/FINAL	N° benign FSE	N° malignant FSE	N° benign FINAL	N° malignant FINAL	local recurrence	follow-up (years)
Ciftci	2001	51	0	5	46	0	5/5	5	0	15	36	no	7.4
Valla	2001	83	0	52	27	2*	34/34	NR	NR	83	0	no	4.8
Metcalfe	2003	51	1	12	38	0	13/13	13	0	13	38	no	3
Shukla	2004	16	0	13	3	0	10/10	10	0	16†	0	no	7.3
Shukla	2004	3	0	1	2	0	1/1	1	0	3‡	0	no	5-14
Lee	2004	209	NR	1	NR	NR	NR	NR	NR	98	111	NR	NR
Tröbs	2007	24	0	4	19	1*	NR	NR	NR	6	18	no	5

TSS - testis-sparing surgery; RO - radical orchidectomy; FSE - frozen section examination; FINAL - permanent histology; NR - not reported

* incisional biopsy

† teratoma

‡ juvenile granulosa cell tumor

The lesions that are the most amenable to TSS are pre-pubertal teratomas, and similarly to the adult age, simple cysts, epidermoid cysts and Leydig cell tumors.

High reliability of FSE has been reported for the diagnosis of pre-pubertal teratoma and epidermoid cysts.[41] In fact, unlike adult testis tumors, which may exhibit mixed histology, almost all pre-pubertal testis tumors are of pure cell type, and the histological features of benign tumors are so characteristic from those of other pre-pubertal testicular lesions that an incorrect diagnosis is virtually impossible. In addition, the concomitant presence of TIN, which is a very common finding in the adult, has never been documented in parenchyma adjacent to these lesions.[39] Following the experience of Rushton *et al*.,[37] Valla *et al*.[42] and Shukla *et al*.,[44] reported the largest series on pre-pubertal teratomas and epidermoid cysts managed with TSS. After a follow-up of up to 22 years, neither local recurrence nor distant metastasis have been observed in 42 patients altogether.

Significant experience has been gained also for Leydig cell tumors. Compared to the adult age cases, almost all infants and children present with a heralding symptom (i.e. precocious pseudopuberty) due to the uniformly hormonally active status of these lesions, and there have been no known cases of unilateral malignant Leydig cell tumor in the pediatric population.[48]

Shukla *et al*.,[45] also reported on the first case of a seven-day-old boy with a juvenile granulosa cell tumor treated with TSS, who was free of recurrence at five-year follow-up, while Nomomura *et al*.,[49] first performed TSS in an eight-year-old child with a large cell calcifying Sertoli cell tumor with neither local recurrence nor ipsilateral atrophy after five years of monitoring.

Based on all these data, many centers of pediatric surgery have now started to systematically apply TSS as the first option in the elective management of testicular tumors when serum tumor markers are within normal range.[50]

## CONCLUSIONS

The old dogma that equaled diagnosis of any testicular mass to immediate RO has been confuted by the clinical experience accumulated in the last decade. Although no randomized controlled trials supporting the use of TSS versus RO for testicular tumors are available and probably will ever be conducted, due to the low incidence of testicular tumors and the long accrual time, increasing evidence deriving also from well-conducted retrospective outcome studies with considerable follow-up suggests that the organ-sparing approach stands for a viable treatment modality for testicular tumors of different histology in the pediatric and adult population. This applies undisputedly to patients with imperative indications for surgery (i.e. solitary testis or bilateral tumors), and, with still some reserve, also to those with elective ones (i.e. presence of a normal contralateral testis), provided that definitive histology fails to reveal malignancy.

The widespread use of high-frequency scrotal ultrasound has led to a marked increase in the number of incidentally detected and small-sized testicular lesions, which have been shown to be prevalently benign. Frozen section examination, which should always be adopted in TSS, has achieved high accuracy in the intraoperative characterization of testicular masses of any nature. Finally, there is increasing awareness of the potential advantages with regard to cosmesis, fertility and endocrine function, related to preserved testicular parenchyma.

Albeit all these are arguments currently favoring organ-sparing surgery, prospective, cooperative, large-scale studies are eagerly awaited to further qualify TSS as a treatment option to be recommended in the management of benign and, under appropriate conditions, malignant testicular tumors.
